# Hepatitis B core-related antigen serum levels may be a predictor of acute flare of chronic hepatitis B among pregnant women in the immune-tolerant phase of chronic HBV infection after short-course antiviral therapy

**DOI:** 10.1080/21505594.2023.2186335

**Published:** 2023-03-09

**Authors:** Ruyu Liu, Liu Yang, Tingting Jiang, Yao Lu, Lu Zhang, Ge Shen, Shuling Wu, Min Chang, Hongxiao Hao, Leiping Hu, Yuanjiao Gao, Mengjiao Xu, Xiaoxue Chen, Wei Yi, Minghui Li, Yao Xie

**Affiliations:** a Department of Hepatology Division 2, Beijing Ditan Hospital, Capital Medical University, Beijing, China; b Department of Obstetrics and gynecology, Beijing Ditan Hospital, Capital Medical University, Beijing, China

**Keywords:** Hepatitis B virus, hepatitis B core-related antigen, acute flare of chronic hepatitis B, short-course antiviral therapy, immune-tolerant phase, pregnant women

## Abstract

**Background:**

Studies have shown acute flares of chronic hepatitis B (CHB) might be related to immunologic changes that occur during pregnancy. However, the indicators for predicting acute flares of CHB among pregnant women still need further study. We aimed to distinguish the relevance between serum levels of HBcrAg and acute flares of CHB in pregnant women in the immune-tolerant phase of chronic HBV infection after short-course antiviral therapy.

**Methods:**

A total of 172 chronic HBV-infected pregnant women who were judged to be in the immune-tolerant phase were recruited in our research. All patients received short-course antiviral therapy with TDF. The biochemical, serological, and virological parameters were measured using standard laboratory procedures. The serum levels of HBcrAg were tested by ELISA.

**Results:**

Fifty-two (30.2%) out of 172 patients had acute flares of CHB. At postpartum week 12 (TDF cessation), serum HBcrAg (OR, 4.52; 95% CI, 2.58–7.92) and HBsAg (OR, 2.52; 95% CI, 1.13–5.65) were associated with acute flares of CHB. The serum HBcrAg levels were beneficial for confirmation of patients with acute flares of CHB, with an area under the ROC curve of 0.84 (95% CI, 0.78–0.91).

**Conclusions:**

For pregnant women with chronic HBV infection in the immune-tolerant phase, serum HBcrAg and HBsAg levels at postpartum week 12 were associated with acute flares of CHB after short-course antiviral therapy with TDF. The serum HBcrAg level can correctly identify acute flares of CHB and may be a predictor of the need for continuing antiviral therapy after 12 weeks postpartum.

## Introduction

The global incidence of hepatitis B s-antigen (HBsAg) is expected to 3.9%, corresponding to 291,992,000 infections [1]. The prevalence of HBsAg is 7.20% in China [1], which increased to 8.16% in women of childbearing age [2]. In view of the WHO’s goal of eliminating viral hepatitis as a major public health threat by 2030, reducing mother-to-child transmission of hepatitis B virus (HBV) through universal infant vaccination and HBV immunoglobulin injection of newborns of HBV-infected women has been a priority for the prevention of HBV infection [3]. Furthermore, for pregnant women with high HBV DNA levels (>200,000 IU/ml), antiviral therapy was also recommended to decrease the chance of mother-to-child transmission [3,4]. However, despite these efforts to reduce mother-to-child transmission of HBV, understanding of the course of chronic HBV infection in pregnant women is still limited.

Previous researches have shown liver aminotransferase levels are apparently increased in about 50% of pregnant women with chronic HBV-infection, mainly occurred in the postpartum period within the first 24 weeks after delivery [5,6]. If alanine aminotransferase (ALT) increased to a certain level, it was considered as an acute flare of chronic hepatitis B. The definition of acute flare of chronic hepatitis B (CHB) lacks consensus; however, it is consistently accepted that the diagnosis of acute flare of CHB must include an increase in ALT levels. Most studies define acute flare of CHB as ALT to 2 times the upper normal limit [5,7,8]. Several studies define acute flare of CHB as ALT to at least 3 times the baseline [9,10]. Acute flare of CHB might be associated with immunologic changes during pregnancy and could give rise to liver injury, hepatic decompensation [11]. A study showed that baseline HBV DNA levels, ALT, hepatitis B e-antigen (HBeAg) status, gravida, age, and parity were not identified as predictors of acute flares of CHB [5,12]. The indicators for predicting acute flares of CHB among pregnant women still need further study.

Current therapies rarely achieve a cure of chronic hepatitis B due to the refractory nature of an intracellular viral replication intermediate termed covalently closed circular (ccc) DNA, which resides in the nucleus of infected cells as an episomal (ie, non-integrated) plasmid-like molecule that gives rise to progeny virus [13]. HBeAg, p22cr (a 22 kDa precore protein), and HBcAg can be classified as hepatitis B core-related antigen (HBcrAg) [14]. The serum concentration of HBcrAg is correlated with intrahepatic cccDNA as well as serum HBV DNA [15]. In immunosuppressed patients, HBcrAg could reflect high levels of HBV DNA and HBV reactivation [16] as well as can also predict HBeAg seroconversion [17]. Moreover, previous researches reported that serum HBcrAg had a bearing on risk of hepatocellular carcinoma [18]. However, there remains limited knowledge about whether serum HBcrAg can predict acute flares of CHB. In the study, we were trying to identify the relationship between HBcrAg serum levels and acute flares of CHB among pregnant women in the immune-tolerant phase of chronic HBV infection after short-course antiviral therapy.

## Methods

### Patients

Guidelines recommend tenofovir disoproxil fumarate (TDF) for pregnant women who require antiviral therapy [3,4,19,20]. In this study, we enrolled 172 pregnant chronic HBV-infected women who underwent short-course antiviral therapy with TDF from January 2019 to April 2022 at Beijing Ditan Hospital, Beijing, China. All enrolled pregnant patients were judged as chronic HBV infection and to be in the immune-tolerant phase [19]. According to Chinese guidelines [19], all enrolled patients received antiviral therapy at 24 weeks of gestation and discontinued TDF at postpartum week 12. In addition, all enrolled patients had normal alanine aminotransferase at 24 weeks gestation. At baseline (24 weeks of gestation) and at different follow-up points, the virological, serological, and biochemical parameters were tested. The exclusion criteria were as follows: (1) coinfection with HIV or hepatitis C, D virus [21–24]; (2) hepatocellular carcinoma patients [25]; (3) treatment with liver-protecting drugs (*N*-acetylcysteine, glutathione, glycyrrhizin acid preparation, bicyclol, polyene phosphatidylcholine, and silymarin) [26] patients; (4) immunosuppressive treatment (glucocorticoids, cyclosporine-A, tacrolimus, mycophenolate mofetil, azathioprine, sirolimus, everolimus); and (5) liver disease caused by reasons other than HBV infection [27–37]. The Institutional Review Board of Beijing Ditan Hospital approved this study (Reference number 2019-003-01), and all enrolled patients signed informed consent forms. A total of 172 patients’ peripheral blood samples of different test points were separated and stored in the biobank’s −80°C refrigerators of Clinical Resources of Beijing Ditan Hospital, Capital Medical University.

### Laboratory data

The standard laboratory procedures were used to detect the virological, biochemical, and serological parameters. All relevant tests were performed by the laboratory department of our hospital. Commercial kits (Abbott Laboratories; Lake Bluff, IL, USA) were used to test serum HBsAg. COBAS TaqMan HBV Test v2.0 (Roche Diagnostics, Branchburg, NJ, USA) was used to assess serum HBV DNA levels. A serum level of 40 IU/mL and 35 IU/mL was set as the upper normal limit of ALT and AST, respectively. A serum HBV DNA level of less than 20 IU/mL was defined as negative.

### Acute flare of CHB assessment

At baseline (24 weeks of gestation), all enrolled patients had normal alanine aminotransferase at 24 weeks of gestation. According to previous studies [5,7,8], ALT to 2 times the upper limit of the normal range (ULN) was defined as acute flare of CHB. If patients develop hepatitis flares, antiviral therapy is indicated [19]. The enrolled 172 patients were assessed independently by 2 clinicians (1 hepatologist and 1 obstetrician). Each patient with acute flare of CHB needs to exclude viral hepatitis not caused by HBV, drug-induced liver injury, alcoholic liver disease, cholestasis of pregnancy, acute fatty liver of pregnancy, HELLP syndrome, and other related diseases with elevated ALT. Discordant patients were assessed by a third expert who was a highly experienced hepatologist.

### Serum HBcrAg detection

Human HBcrAg ELISA Kit (Jianglai Industrial Limited by Share Ltd, Shanghai, China) was used to measure serum levels of HBcrAg [38]. The detection of the samples to be tested was carried out according to the experimental steps of the kit. Briefly, anti-HBcrAg antibodies were precoated in microtiter wells. The microtiter wells for standard sample and the samples under test were arranged on the microtiter plates. Different concentrations of standard samples were added to the standard sample microtiter wells, and the samples to be tested were also added to the corresponding microtiter wells. After that, a detection antibody conjugated to horseradish peroxidase was added to each well. The microtiter plates were incubated in an incubator at 37°C for 60 min. After washing five times with wash buffer, tetramethylbenzidine (TMB) chromogen solution A and B were added to each microtiter well, respectively, and the microtiter plates were incubated again in the incubator at 37°C for 15 min under darkness. After incubation, stop solution was added to each well. Finally, microtiter plate reader (Varioskan Flash, Thermo, USA) was used to measure the optical density (OD) value of each well at 450 nm within 15 min.

### Statistical analysis

SPSS version 22.0 (SPSS, Inc., Chicago, IL, USA) was used for statistical analysis. The normally distributed data and nonnormally distributed continuous data were presented as the mean ± SD, median (interquartile range), respectively. The significance of differences between different groups was performed by the *t* test or Mann–Whitney test. The correlation between virological, biochemical, and serological parameters and acute flare of CHB was conducted by logistic regression analysis. SPSS version 22.0 was used to calculate the area under the receiver operating characteristic curve (AUROC) with 95% confidence interval (CI). Single asterisk (*) represents *p* value <0.05 and double asterisks (**) represent *p* value <0.01.

## Results

### Characteristics of patients at baseline and different points after delivery

The characteristics of patient dataset are presented in Table 1. All 172 patients were HBeAg positive. Compared with baseline, ALT and AST increased significantly at postpartum week 12, postpartum week 16, postpartum week 24, and postpartum week 36 (*p* < 0.01). The WBC count at delivery was significantly higher than that at baseline and at other follow-up points after delivery (*p* < 0.01). RBC, HGB, PLT, and ALB levels at postpartum week 12, postpartum week 16, postpartum week 24, and postpartum week 36 were significantly higher than those at baseline and the delivery point (*p* < 0.01), respectively. TBIL, DBIL, PTA, and HBsAg were not significantly different between baseline and postpartum week 12, postpartum week 16, postpartum week 24, and postpartum week 36 (*p* > 0.05).

**Table 1.** Characteristics of chronic HBV-infected pregnant patients.

Characteristics	Baseline (24 weeks of gestation)	At delivery	Postpartum week 12	Postpartum week 16	Postpartum week 24	Postpartum week 36
Age (y, M ± SD)	26.49 ± 3.77	-	-	-	-	-
WBC count (10^9^/L)	6.38 ± 1.45	8.42 ± 2.33**	6.42 ± 1.36	6.65 ± 1.55	6.26 ± 1.31	6.19 ± 1.24
RBC count (10^12^/L)	4.13 ± 0.42	4.01 ± 0.36	4.64 ± 0.53**	4.75 ± 0.56**	4.91 ± 0.60**	4.73 ± 0.56**
HGB (g/L)	118.63 ± 12.3	116 ± 3.28	125.74 ± 16.47**	127.96 ± 17.31**	133.02 ± 16.02**	138.17 ± 16.93**
Platelet count (10^9^/L)	208.05 ± 57.30	205 ± 55.47	225.9 ± 62.51**	229.52 ± 59.86**	237.17 ± 59.05**	241.63 ± 59.65**
ALT, U/L (median)	20.0 (15.4–29.35)	22.74(16.3–26.53)	32.4 (20.33–58.78)**	42.3 (32.4–67.9)**	33.1 (21.3–60.15)**	29.32(19.56–43.17)**
AST, U/L (median)	21.1 (17.95–25.55)	21.8(18.01–26.66)	24.65 (19.75–30.57)**	32.1 (22.18–49.2)**	25.23(19.56–33.29)**	25.05 (20.95–30.13)**
TBIL, (µmol/L, M ± SD)	9.91 ± 2.94	9.82 ± 2.58	10.44 ± 3.49	10.97 ± 3.26	10.46 ± 3.34	10.38 ± 3.69
DBIL, (µmol/L, M ± SD)	3.19 ± 1.73	3.11 ± 1.68	3.07 ± 1.75	3.62 ± 1.96	3.33 ± 2.42	3.59 ± 2.11
ALB, (g/L, M ± SD)	38.7 ± 4.45	35.49 ± 3.14	41.91 ± 4.44**	41.79 ± 2.33**	43.12 ± 3.21**	45.34 ± 3.22**
PTA (M ± SD)	113.38 ± 13.69	112.71 ± 12.98	110.81 ± 13.17	111.68 ± 12.36	113.22 ± 12.38	114.19 ± 13.46
HBsAg (log10) (median)	4.44 (3.73–4.60)	4.67(3.54–4.72)	4.35 (3.23–4.57)	4.17 (3.02–4.46)	4.51 (3.48–5.07)	4.26 (3.67–4.72)
HBeAg positive/negative	172/0	172/0	172/0	172/0	172/0	172/0
HBcrAg (M ± SD)	0.47 ± 0.19	0.31 ± 0.19	0.30 ± 0.18	0.42 ± 0.16	0.45 ± 0.15	0.46 ± 0.17
HBV-DNA (log10) (median)	8.08 (5.89–8.23)	4.71 (3.11–6.41)	3.66(2.31–4.27)	3.43 (2.14–4.05)	7.84 (7.1–8.24)	8.12(5.93–8.31)

Note: **p < 0.01.

### Acute flare of CHB among pregnant women after short-course antiviral therapy

In the light of the prevention and treatment of CHB of Chinese guidelines (2019 version), all patients treated with TDF discontinued therapy at postpartum week 12. Eighty (46.5%) out of 172 patients had elevated ALT levels, and 52 (30.2%) out of 172 patients had acute flares of CHB. The incidence of acute flares of CHB during pregnancy was 12.2% (21/172), while it was 18% (31/172) after delivery. Forty-nine (94.2%) out of 52 patients’ ALT levels were within 5 times the ULN, and only 3 (5.8%) patients’ ALT levels were more than 5 times the ULN (Table 2). All of the acute flares of CHB patients resumed TDF therapy, and the alanine aminotransferase flares resolved gradually (Table 3).

**Table 2.** ALT and HBV DNA levels of acute flare of CHB patients.

Variable	Number
ALT levels (U/L)	
1.1 to 2× ULN	28
2.1 to 5× ULN	49
>5× ULN	3
Acute flares of CHB	
During pregnancy	21
24 weeks – delivery	21
After delivery	31
0–12 weeks after delivery	24
13–24 weeks after delivery	6
25–36 weeks after delivery	1
HBVDNA (IU/mL)	
>2000	47
20–2000	5

**Table 3.** Dynamic changes in ALT and AST at different test points.

Characteristics		Baseline	At delivery	Postpartum week 12	Postpartum week 16	Postpartum week 24	Postpartum week 36
ALT, U/L (median)	Acute flare of CHB patients	20.3 (13.52–32.45)	54.6(38.35–73.9)	60.2(52.05–85.2)	70.1(53.1–95.65)	48.6(43.9–67.4)	42.5(28.53–55.53)
	No acute flare of CHB patients	20.0 (16.55–26.55)	19.3(14.65–25.25)	21.5(14.85–26.1)	19.9(12.5–26.65)	21.2(16.05–26.9)	19.6(14.1–26.0)
AST, U/L (median)	Acute flare of CHB patients	21.25 (18.2–27.38)	52.4(20.8–92.35)	59.1(52.05–76.5)	64(51.53–82.03)	38.9(27.4–54.1)	34.7(27–42.5)
	No acute flare of CHB patients	20.6(18.05–23.7)	20.3(17.7–26.1)	21.4(17.23–26.88)	20.55(16.45–25.28)	22.3(19.48–28.55)	19.9(18–24.1)

### Incidence of HBV DNA increase and hepatic decompensation

The serological, virological, and biochemical parameters were determined at baseline, delivery point, postpartum week 12, postpartum week 16, postpartum week 24, and postpartum week 36. The HBV DNA levels of the enrolled patients gradually decreased after the initiation of TDF therapy (Table 1). However, when the drug was discontinued (postpartum week 12), HBV DNA levels gradually increased again (Table 1). Among the 52 patients confirmed as acute flare of CHB with ALT ≥ 2 times ULN, 47 patients had HBV DNA >2000 IU/mL, and 5 patients had HBV DNA between 20 and 2000 IU/mL at postpartum week 12 (Table 2). Through analysis of biochemical, serological, and virological indicators, none of the patients developed hepatic decompensation.

### None of the infants developed mother-to-child transmission

**Figure 1.**
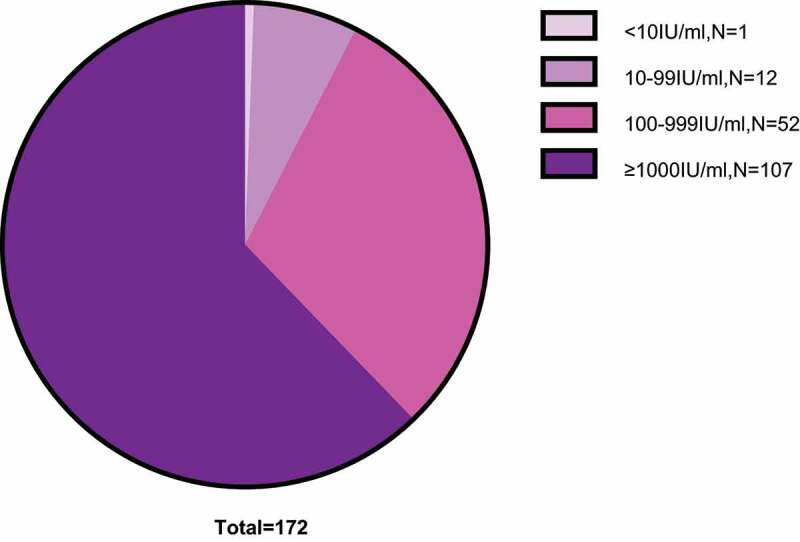
Characteristics of newborn infants of chronic HBV-infected mothers.

All 172 mothers gave birth to 172 infants. The characteristics of all 172 infants are shown in Table 3. Ninety-four of 172 infants were male, and 78 were female. All the infants were given 200 IU of hepatitis B immune globulin intramuscularly at birth, 10 μg of HBV vaccine within 6 h after birth, and two additional vaccinations at week 4 and 24 [39, 40]. No infants were HBsAg-positive at week 28. A total of 171 of 172 infants were HBsAb positive, and 1 infant was HBsAb negative at week 28. Among these 171 HBsAb-positive infants, the numbers in different HBsAb titer groups were as follows: 12 in the 10–99 IU/ml group, 52 in the 100–999 IU/ml group, and 107 in the ≥1000 IU/ml group (Figure 1).

A total of 171 of 172 infants were HBsAb positive, and 1 infant was HBsAb negative at week 28. Among the 171 infants, 12 in the 10–99 IU/ml group, 52 in the 100–999 IU/ml group, and 107 in the ≥1000 IU/ml group.

### Serum HBcrAg levels increased significantly after TDF cessation

**Figure 2.**
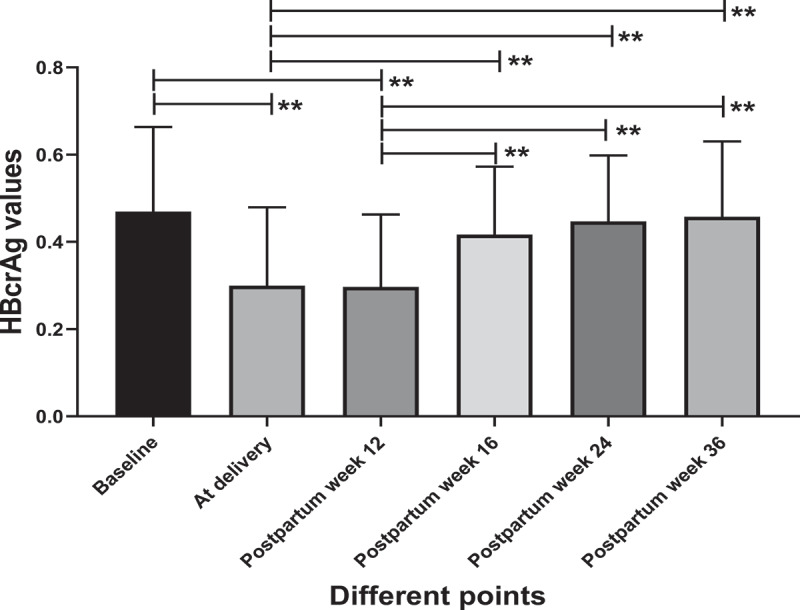
Dynamic changes in HBcrAg at different test points.

Compared with baseline, serum HBcrAg levels were significantly decreased at delivery and postpartum week 12 (TDF cessation) after TDF antiviral treatment (*p* < 0.01) (Figure 2). We compared the serum HBcrAg levels between postpartum week 12 and postpartum week 16, postpartum week 24 and postpartum week 36, and HBcrAg increased significantly (*p* < 0.01) (Figure 2). The serum HBcrAg levels were also compared between the delivery and postpartum week 16, postpartum week 24 and postpartum week 36. The HBcrAg also significantly increased (*p* < 0.01), but there was no significant difference among these three test points (Figure 2). The serum HBcrAg levels showed no significant difference between delivery and postpartum week 12 (*p* > 0.05) (Figure 2).

Compared with baseline, HBcrAg levels significantly decreased at delivery and postpartum week 12 (TDF cessation) after TDF antiviral treatment (**p < 0.01). After TDF discontinuance, HBcrAg increased significantly at postpartum week 16, postpartum week 24, and postpartum week 36. Compared with delivery, HBcrAg levels significantly increased at postpartum week 16, postpartum week 24, and postpartum week 36 (**p < 0.01).

### HBcrAg increased significantly in acute flare of CHB patients at postpartum week 12

**Figure 3.**
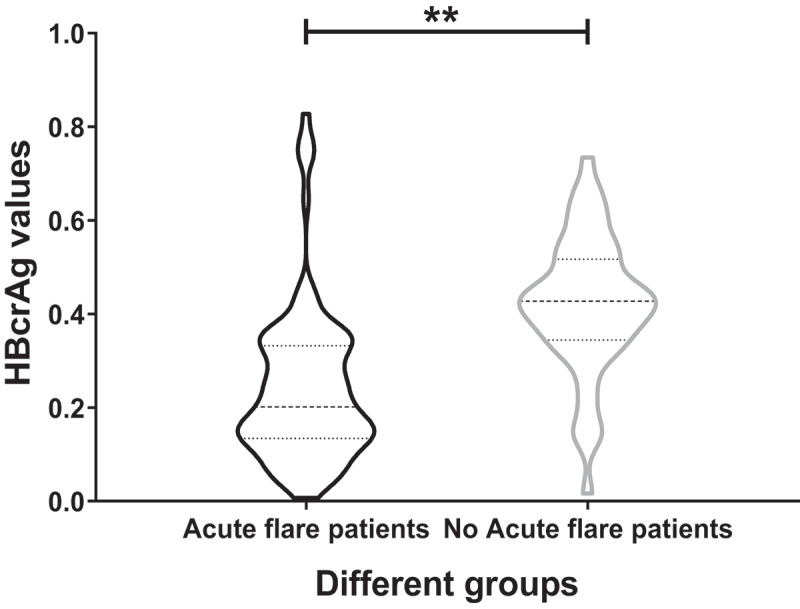
HBcrAg levels of acute flare of CHB patients and no acute flare of CHB patients at postpartum week 12.

In postpartum week 12, TDF therapy was stopped, and the serum HBcrAg values were tested. As we showed above, 52 out of 172 patients had acute flares of CHB. We compared the serum HBcrAg levels between the acute flare of CHB patients (52) and the no acute flare of CHB patients (120) at this point. The data analysis showed that the serum HBcrAg levels raised remarkably in the acute flare of CHB patients (*p* < 0.01) (Figure 3).

Compared with no acute flare of CHB patients, HBcrAg levels significantly increased in the acute flare of CHB patients at postpartum week 12 (**p < 0.01).

### Serum HBcrAg values at postpartum week 12 can identify acute flares of CHB

**Figure 4.**
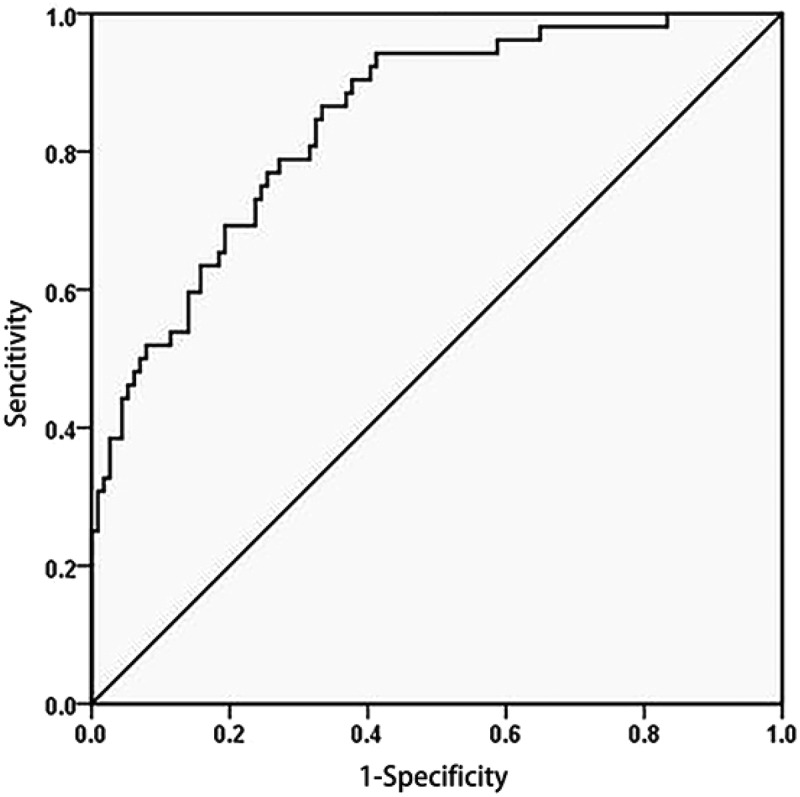
ROC curve analysis of serum HBcrAg in identifying patients with acute flares of CHB.

A ROC curve was used to analyse the efficiency of the serum HBcrAg level in the diagnosis of acute flares of CHB. The serum HBcrAg value at postpartum week 12 can correctly identify patients with acute flares of CHB, with an AUROC of 0.84 (95% CI, 0.78–0.91) (Figure 4), and the cut-off value was ≥0.275 for the Youden index. A HBcrAg level <0.275 showed a negative predictive rate of 92.01% for excluding acute flares of CHB, and the sensitivity and specificity were 86.5% and 67.5%, respectively (Table 4).

The HBcrAg serum value can effectively identify patients with acute flares of CHB, with an AUROC of 0.84 (95% CI, 0.78–0.91).

**Table 4.** Negative predictive value of serum HBcrAg in predicting acute flares of CHB.

	Hepatitis B		
HBcrAg	No acute flare of CHB	Acute flare of CHB	Total
<0.275	85.7% (*n* = 81)	14.3% (*n* = 7)	88
≥0.275	28.3% (*n* = 39)	71.7% (*n* = 45)	84
Total	120	52	172

### HBcrAg and HBsAg were associated with acute flares of CHB

The correlation between acute flares of CHB and WBC, RBC, HGB, PLT, HBsAg, HBV DNA, HBcrAg, ALT, AST, TBIL, ALB, and PTA were analysed by logistic regression analysis. Analysis results showed that HBcrAg serum levels (OR, 4.52; 95% CI, 2.58–7.92) and HBsAg serum levels (OR, 2.52; 95% CI, 1.13–5.65) at postpartum week 12 were associated with acute flares of CHB (Table 5). These two indicators are independent risk factors for acute flares of CHB, while the levels of WBC, RBC, HGB, PLT, ALT, AST, TBIL, ALB, PTA, and HBV DNA were not associated with acute flares of CHB.

**Table 5.** HBcrAg and HBsAg are associated with acute flare of CHB.

Factors	OR	95% CI	*P* value
ALT	1.41	0.80–2.47	0.234
AST	1.49	0.85–2.60	0.163
TBIL	1.09	0.70–1.68	0.712
ALB	0.82	0.53–1.26	0.367
PTA	0.79	0.52–1.19	0.255
HBsAg	2.52	1.13–5.65	0.025*
HBV DNA	1.22	0.72–2.06	0.457
HBcrAg	4.52	2.58–7.92	<0.001**
WBC	1.14	0.76–1.79	0.536
RBC	1.55	0.91–2.63	0.109
HGB	0.69	0.41–1.16	0.164
PLT	1.01	0.65–1.59	0.962

Note: *p < 0.01, **p < 0.01.

## Discussion

HBcrAg has been regarded as one of the serum markers for assessment of cccDNA activity [15,41]. Serum HBcrAg levels of HBeAg-positive patients were noticeably higher than HBeAg-negative patients [17]. Elevated serum HBcrAg in HBV-infected patients greatly strengthens the risk of progression to cirrhosis, and serum HBcrAg is considered as a good predictor for the development of cirrhosis [42]. Researchers also established that HBcrAg level was involved in progression of liver fibrosis, particularly in CHB patients treated with nucleoside analogs [43]. More studies are needed to clarify whether HBcrAg is a serum biomarker of acute flares of CHB for chronic HBV-infected pregnant patients who underwent short-course antiviral therapy by TDF.

Here, 172 chronic HBV-infected pregnant patients were enrolled and underwent short-course antiviral therapy with TDF and discontinued it at postpartum week 12. All pregnant patients were judged to be in the immune-tolerant phase. The correlation between acute flares of CHB and virological, serological, and biochemical parameters of these patients was analysed by logistic regression analysis. We found that serum levels of HBcrAg at postpartum week 12 were associated with acute flares of CHB and were an independent risk factor for acute flares of CHB. Effective identification of patients with acute flares of CHB was realized by detection of serum HBcrAg. A HBcrAg level <0.275 showed a negative predictive rate of 92.01% to exclude acute flares of CHB, with a sensitivity of 86.5% and a specificity of 67.5%. Previous study has shown that delaying drug withdrawal might delay the onset of postpartum hepatitis [44]. Based on our findings, continued antiviral therapy with TDF is recommended for patients with serum HBcrAg level ≥0.275.

We also found that serum levels of HBsAg at postpartum week 12 were associated with acute flares of CHB. Previous studies have shown that HBsAg at the end of therapy can predict off‐therapy relapse [45–47], which includes clinical relapse that was defined as serum ALT greater than twofold ULN [47]. Previous research reported that serum levels of HBsAg were associated with clinical relapse, and the hazard ratio (per 23 log IU/mL increment) for clinical relapse was 2.47 (95% CI, 1.45–4.23) [45]. Our results are consistent with these previous reports [45–47].

In our study, all the patients treated with TDF discontinued therapy at postpartum week 12 based on Chinese guidelines (2019 version). Mother-to-child transmission was successfully eliminated, and none of the newborn infants developed chronic HBV infection. Eighty (46.5%) out of 172 patients had elevated ALT levels, and 52 (30.2%) out of 172 patients had acute flares of CHB. The incidence of acute flares of CHB was higher in the postpartum period than pregnancy period, similar to previous reports [6]. Among the patients diagnosed as acute flare of CHB, 90.4% had HBV DNA >2000 IU/mL. No patients developed hepatic decompensation.

We tested serum HBcrAg levels after TDF therapy and TDF discontinuance at different follow-up points. Serum HBcrAg levels were significantly decreased at delivery and postpartum week 12 (TDF cessation) after TDF antiviral treatment (*p* < 0.01). However, after TDF discontinuance, the serum HBcrAg increased significantly at postpartum week 16, postpartum week 24 and postpartum week 36 (*p* < 0.01). A study in Japan achieved similar results [48]. We further tested serum HBcrAg values in acute flare of CHB patients and no acute flare of CHB patients at postpartum week 12. We found that the serum HBcrAg levels significantly increased in the acute flare of CHB patients (*p* < 0.01).

This study also has limitations. First, only pregnant patients with chronic HBV infection in the immune-tolerant phase were studied in this work, and patients with chronic HBV infection in other phases also need to be further studied. Second, chemiluminescent immunoassay was performed to determine serum HBcrAg. Currently, Lumipulse HBcrAg assay (Fujirebio, Japan) is the most widely used commercial assay and has been validated for quantifying HBcrAg levels. In the measurement of serum HBcrAg in our study, our kit was not validated against Lumipulse HBcrAg assay, so its reliability needs further investigation. Third, the low number of patients involved in the study was another limitation. We will enrol more patients who meet the enrolment criteria in subsequent studies to further investigate the association between hepatitis B core-related antigen serum levels and acute flare of chronic hepatitis B.

## Conclusions

In this study, 30.2% of enrolled chronic HBV-infected postpartum women who received short-course antiviral therapy developed pregnancy-associated acute flares of CHB. For pregnant women with chronic HBV infection in the immune-tolerant phase, serum HBcrAg and HBsAg levels at postpartum week 12 (TDF cessation) were associated with acute flares of CHB after they underwent short-course antiviral therapy with TDF, and these two indicators are independent risk factors for acute flares of CHB. The serum HBcrAg level can correctly identify acute flares of CHB and may be a predictor of the need for continuing antiviral therapy after 12 weeks postpartum.

## Supplementary Material

Supplemental Material

## Data Availability

The datasets used and/or analysed during the current study are available from the corresponding author upon reasonable request. If you need supporting data, you can contact us at any time. Email: wuhm2000@sina.com.

## Notes

Here are several current universal guidelines and their recommendations, which we compared with the Chinese guidelines. They are as follows:

**Asian Pacific Association for the Study of the Liver (APASL) guidelines: hepatitis B virus in pregnancy (2022 version)**: The APASL suggests pregnant females testing HBsAg positive with an HBV DNA ≥200,000 IU/ml should receive antiviral prophylaxis for prevention of mother-to-child transmission of HBV. The antiviral therapy should be initiated at 24–28 weeks of gestation, and tenofovir disoproxil fumarate (TDF) is recommended. Pregnant females taking antivirals only can discontinue antiviral treatment immediately after delivery or continue up to 12 weeks postpartum and should be monitored closely for hepatitis flare and rebound of HBV DNA till at least 24 weeks. Antiviral treatment can be considered if meeting treatment indications for therapy [20].

**Update on Prevention, Diagnosis, and Treatment of Chronic Hepatitis B AASLD 2018 Hepatitis B Guidance**: The AASLD suggests antiviral therapy to reduce the risk of perinatal transmission of HBV in HBsAg-positive pregnant women with an HBV DNA level >200,000 IU/ml, and antiviral therapy should start at week 28–32 of gestation. TDF is preferred to minimize the risk of emergence of viral resistance during treatment. Antiviral therapy was discontinued at birth to 12 weeks postpartum in most of the studies. With discontinuation of treatment, women should be monitored for ALT flares every 12 weeks for 24 weeks. For pregnant women with immune-active hepatitis, treatment should be based on recommendations for nonpregnant women [3].

**EASL 2017 Clinical Practice Guidelines on the management of hepatitis B virus infection**: The EASL suggests that in all pregnant women with HBV DNA levels >200,000 IU/ml or HBsAg levels >4 log10 IU/ml, antiviral prophylaxis with TDF should start at week 24–28 of gestation and continue for up to 12 weeks after delivery [4].

**Chinese guidelines for the prevention and treatment of chronic hepatitis B (2019 version)**: The Chinese guidelines suggest pregnant women with an HBV DNA levels >200,000 IU/ml should receive antiviral prophylaxis with TDF or telbivudine. The antiviral therapy should start at week 24–28 of gestation. Pregnant women who received antiviral therapy with TDF or telbivudine during the immune tolerance period can discontinue TDF or telbivudine immediately after delivery or continue for another 4–12 weeks. After drug withdrawal, postpartum women should be monitored closely for hepatitis fare and rebound of HBV DNA for 24 weeks. If hepatitis flare occurred after delivery, antiviral therapy is recommended [19].

The APASL, AASLD, and EASL guidelines are universal guidelines [3,4,20]. For pregnant chronic hepatitis B patients with HBV DNA levels >200,000 IU/ml, antiviral therapy with TDF is recommended by these three guidelines and Chinese guidelines; in addition, the Chinese guidelines also recommend the use of telbivudine for antiviral therapy [19]. The AASLD and APASL recommend discontinuing antiviral treatment immediately after delivery or continuing up to 12 weeks postpartum. EASL guidelines recommend antiviral therapy continuing for up to 12 weeks after delivery. However, Chinese guidelines recommend that pregnant women taking TDF or telbivudine at the immune tolerance phase discontinue antiviral treatment immediately after delivery or continue for another 4–12 weeks. The EASL, APASL,and Chinese guidelines suggest that antiviral therapy should start at week 24–28 of gestation, while AASLD guidelines recommend to start antiviral therapy at week 28–32 of gestation (Table S1).

The AASLD, APASL, and Chinese guidelines suggest that women with discontinuation of treatment should be monitored for hepatitis flare for 24 weeks. APASL guidelines suggest antiviral treatment can be considered if meeting treatment indications for therapy. Chinese guidelines recommend antiviral therapy in cases of hepatitis flares after delivery. The AASLD and EASL guidelines have no specific treatment recommendations for hepatitis flares after delivery (Table S1).
