# Supplementary material for: Hepatitis B core-related antigen serum levels may be a predictor of acute flare of chronic hepatitis B among pregnant women in the immune-tolerant phase of chronic HBV infection after short-course antiviral therapy
Source: Virulence. 2023 Mar 9;14(1):2186335. doi: 10.1080/21505594.2023.2186335 (PMC10012896; doi:10.1080/21505594.2023.2186335)
Supplement: Supplemental Material [file KVIR_A_2186335_SM5570.docx]

Supplementary Table 1. Similarities and differences among the four guidelines recommended for pregnant women infected with HBV.

| Characteristics | APASL (2022 version) | AASLD (2018 version) | EASL (2017 version) | China (2019  version) |
| --- | --- | --- | --- | --- |
| HBVDNA levels need to be treated (IU/ml) | ≥200,000 | >200,000 | >200,000 | >200,000 |
| Antiviral drugs | TDF | TDF | TDF | TDF or telbivudine |
| Discontinuation points of treatment | After delivery or continue up to 12 weeks postpartum | After delivery or continue up to 12 weeks postpartum | Continue for up to 12 weeks postpartum | After delivery or continue for another 4 to12 weeks |
| Time to start antiviral therapy | week 24–28 of gestation | week 28–32 of gestation | week 24–28 of gestation | week 24–28 of gestation |
| Monitoring duration after delivery | 24 weeks | 24 weeks | No recommendation | 24 weeks |
| Treatment recommendation | Antiviral treatment can be considered if meeting treatment indications for therapy | No recommendation | No recommendation | antiviral therapy is recommended if hepatitis flare occurred after delivery |
